# Adult food choices depend on sex and exposure to early-life stress: Underlying brain circuitry, adipose tissue adaptations and metabolic responses

**DOI:** 10.1016/j.ynstr.2021.100360

**Published:** 2021-06-28

**Authors:** S.R. Ruigrok, J.M. Kotah, J.E. Kuindersma, E. Speijer, A.A.S. van Irsen, S.E. la Fleur, A. Korosi

**Affiliations:** a Center for Neuroscience, Swammerdam Institute for Life Sciences, University of Amsterdam, Amsterdam, the Netherlands; b Amsterdam UMC, University of Amsterdam, Laboratory of Endocrinology, Department of Clinical Chemistry & Department of Endocrinology & Metabolism, Amsterdam Neuroscience, Meibergdreef 9, Amsterdam, Netherlands; c Netherlands Institute for Neuroscience, An Institute of the Royal Netherlands Academy of Arts and Sciences (KNAW), Meibergdreef 47, Amsterdam, Netherlands

**Keywords:** Early-life stress, Food choice, Metabolism, Sex differences, Reward circuitry

## Abstract

Exposure to early-life stress (ES) increases the risk to develop obesity later in life, and these effects may be sex-specific, but it is currently unknown what underlies the ES-induced metabolic vulnerability. We have previously shown that ES leads to a leaner phenotype under standard chow diet conditions, but to increased fat accumulation when exposed to an unhealthy obesogenic diet. However these diets were fed without a choice. An important, yet under investigated, element contributing to the development of obesity in humans is the choice of the food. There is initial evidence that ES leads to altered food choices but a thorough testing on how ES affects the choice of both the fat and sugar component, and if this is similar in males and females, is currently missing. We hypothesized that ES increases the choice for unhealthy foods, while it at the same time also affects the response to such a diet. In a mouse model for ES, in which mice are exposed to limited nesting and bedding material from postnatal day (P)2–P9, we investigated if ES exposure affected i) food choice with a free choice high-fat high-sugar diet (fcHFHS), ii) the response to such a diet, iii) the brain circuits that regulate food intake and food reward and iv) if such ES effects are sex-specific. We show that there are sex differences in food choice under basal circumstances, and that ES increases fat intake in females when exposed to a mild acute stressor. Moreover, ES impacts the physiologic response to the fcHFHS and the brain circuits regulating food intake in sex-specific manner. Our data highlight sex-specific effects of ES on metabolic functioning and food choice.

## Highlights

• Strong sex differences exist in food choice and metabolism in mice.
• Early-life stress (ES) increases fat intake in females after mild acute stress exposure.
• The physiological response to the diet is affected by ES in a sex-dependent manner.
• ES modulates the hedonic feeding circuitry.

## Abbreviations

ES: Early-life stress
CTL: Control
fcHFHS: Free choice high-fat high-sugar diet
STD: Standard diet
CORT: Glucocorticoids
AgRP: Agouti-related protein
TH: Tyrosine hydroxylase
GAD65: Glutamate decarboxylase 65
ARH: Arcuate nucleus of the hypothalamus
VTA: Ventral tegmental area
NAcc: Nucleus accumbens
CD36: Cluster of differentiation 36
FABP4: Fatty acid binding protein 4
FASN: Fatty acid synthase
PPARγ: Peroxisome proliferator-activated receptor gamma

## 1 Introduction

Metabolic diseases are increasingly common in the modern society in which high caloric foods are abundant and readily available (Malik et al., 2013). In 2016, worldwide 39% of adults were overweight and 13% were obese (World Health Organization, 2018). Thus, it is key to understand which factors contribute to the development of obesity. The vulnerability to become overweight or obese is heavily influenced by the perinatal environment, during which a metabolic setpoint is likely established (Bouret, 2009; Bouret and Simerly, 2006; Levin, 2006). There is evidence that exposure to early-life adversity increases the risk to develop metabolic diseases, including obesity and diabetes, later in life (Alciati et al., 2013; Balsevich et al., 2019; Bellis et al., 2015; Danese and Tan, 2014) and these effects are potentially sex specific (Boynton-Jarrett et al., 2010; Kozyrskyj et al., 2011; Murphy et al., 2018; Park et al., 2018). Early-life stress (ES) exposure affects many children worldwide. For example, in the United States, it is estimated that 61% of adults experienced some form of ES (e.g. abuse, neglect, parental separation, poverty) (Merrick et al., 2018). It is therefore urgently needed to understand how ES impacts metabolic vulnerability in order to develop strategies to prevent and reduce the incidence of metabolic disorders later in life. Such an early-life-induced setpoint might determine how an organism will respond to its later life nutritional environment, but possibly also the food choices an individual makes in the first place.

To understand ES-induced metabolic vulnerability, it is important to include a metabolic challenge, like the exposure to an unhealthy diet later in life (Maniam et al., 2014; Maniam and Morris, 2010; Murphy et al., 2018; Panetta et al., 2017; Yam et al., 2017a). For example, we have previously reported that, while ES exposure followed by a (healthy) standard chow diet later in life resulted in a leaner phenotype, a western-style diet (WSD) exposure increased adipose tissue accumulation in ES animals compared to controls (Yam et al., 2017a), suggesting an increased vulnerability to such an unhealthy diet. Dietary exposure studies most often use a design with an *ad libitum* availability of the obesogenic diet, without any option for a choice. While such an experimental design allows to study the physiological response to an unhealthy diet, this deviates from the human condition where individuals can make food choices. In fact, a few studies suggest that ES may increase the preference for unhealthy foods, possibly further contributing to the increased metabolic vulnerability. For example, child maltreatment was shown to be a strong predictor of obesogenic food consumption in young children (Jackson and Vaughn, 2019), and prenatal exposure to the Dutch famine has been associated with increased preference for fatty foods in adulthood (Lussana et al., 2008). Moreover, in a study on female rats, ES exposure increased preference for palatable chow, containing both increased fat and sugar levels, over standard chow (Machado et al., 2013), giving a first indication of a direct relationship between ES exposure and food choice, rather than only being circumstantial (i.e. children exposed to ES may more often grow up in poverty situations and/or in environments with higher availability to unhealthy foods (Conroy et al., 2010; Smoyer-Tomic et al., 2008)). Thus, a lot remains to be understood about how ES increases metabolic risk and whether there indeed is a role for food choice.

The regulation of energy homeostasis (e.g. energy intake and expenditure) and food choice is regulated by a complex interplay between the periphery and the brain (Timper and Brüning, 2017). Peripherally, the adipose tissue has a crucial role in energy homeostasis. Besides storing lipids, the white adipose tissue (WAT) is also an active endocrine organ, which secretes adipokines including leptin. In obesity, WAT expands by both increasing adipocyte numbers as well as cell size. Rapid expansion associated with weight gain is accompanied by local inflammation and insulin resistance (Mraz and Haluzik, 2014; Ouchi et al., 2011). Adipose tissue dysfunction is considered to be a determinant metabolic complications associated with obesity (Longo et al., 2019). Interestingly, we have shown before that ES induced by limiting the bedding and nesting material (LBN) affects WAT inflammatory and adipokine gene expression (Ruigrok et al., 2021; Yam et al., 2017a). Centrally, homeostatic as well as reward neural circuitry are at play. The hypothalamus is involved in the homeostatic regulation of food intake, and integrates signals from hormones (e.g. leptin, insulin, ghrelin) and nutrients (e.g. glucose, fatty acids) to determine energy availability (Lam et al., 2005; Timper and Brüning, 2017). The arcuate nucleus of the hypothalamus contains two main neuronal cell populations that do so: agouti-related protein (AgRP) and neuropeptide Y (NPY) expressing neurons which stimulate food intake, and pro-opiomelanocortin (POMC) expressing neurons that inhibit food intake. The mesolimbic pathway is involved in the rewarding aspects of food intake, with dopamine signaling in the ventral tegmental area (VTA) being central to this pathway. For food intake, the connection between the VTA and the striatum including the nucleus accumbens (NAcc) is most studied (Volkow et al., 2011), and it has been shown that palatable foods such as fat and sugar increase dopamine release within the NAcc (De Macedo et al., 2016). While the homeostatic and reward circuits are often studied separately, they are actually integrated at several levels including a connection between the hypothalamus and the VTA (Dietrich et al., 2012; Hsu et al., 2018). It has been shown that AgRP neurons innervate the VTA via GABAergic connections, and AgRP has been shown to function as a reward circuit setpoint by affecting dopamine cell excitability and dopamine levels (Dietrich et al., 2012). Indeed, ablating hypothalamic AgRP increases palatable food intake, and feeding responses in AgRP-ablated mice depended on dopamine tone (Denis et al., 2015). In fact, AgRP ablation has been proposed to create a context favourable of comfort eating in which feeding is less driven by energy demand and more sensitive to stress and reward (Denis et al., 2015). Interestingly, we have earlier reported that ES (LNB model) altered hypothalamic AgRP fiber density early in life (Yam et al., 2017b), and ES induced by maternal separation has been linked to exacerbated food-motivated behaviour, and blunted dopamine release in the NAcc upon consumption of palatable foods (Romaní-Pérez et al., 2016), suggesting that both these circuits might be at play in the effects of ES on food choice and metabolism.

When investigating food choice in rodents, it is important to mimic human dietary choices as much as possible. For such studies, often the fatty and sugar components are combined in the chow, however we have shown earlier that a free choice high-fat high-sugar diet (fcHFHS) consisting of a choice among a solid fatty component (100% beef tallow), a sugary drink (10% sucrose water), a nutritionally complete chow and water, causes prolonged hyperphagia, snacking behaviour and increased food-motivated behaviour in rodents, simulating more closely what is observed in human obesity (la Fleur et al., 2010; La Fleur et al., 2014). In the current study, we set out to test whether ES affects i) food choice using a fcHFHS paradigm, ii) the metabolic response (i.e. adipose tissue mass and gene expression) to such a diet, as well as iii) the underlying brain circuits (i.e. the hypothalamus and VTA), and iv) whether these measures are different in males and females. We show that food choice and the metabolic response to the fcHFHS are different for males and females, that ES exposure affects food choice upon a mild stressor (in the form of tail cuts and 4 h fasting) as well as the metabolic response to the diet in a sex-specific way, and provide evidence that the brain circuits that regulate hedonic food intake are affected by previous ES exposure.

## 2 Materials and methods

### 2.1 Mice and breeding

For these studies, 96 experimental animals (CTL M STD: n = 10; ES M STD: n = 8; CTL F STD: n = 10; ES F STD: n = 13; CTL M HFHS: n = 15; ES M HFHS: n = 11; CTL F HFHS: n = 13; and ES F HFHS: n = 15) were used, originating from 18 litters (9 CTL, 9 ES), each containing five-six pups. Animals were kept under standard housing conditions (temperature 20–22C, 40–60% humidity, 12/12 h light/dark schedule). Standard chow and water *ad libitum* were provided, unless noted otherwise. Animals were weaned at postnatal day 21, and group-housed until the dietary choice experiment started. All experimental procedures were conducted under national law and European Union directives on animal experiments, and were approved by the animal welfare committee of the University of Amsterdam.

Experimental animals were bred in house to standardize the perinatal environment. Eight-ten week old C57Bl/6 J female and male mice were purchased from Invigo Laboratories B.V. (Venray, The Netherlands). After habituation for one-two weeks, two primiparous females were housed together with one adult male to allow for mating, for one week. Females were housed together for another week and were given nesting material (square piece of cotton) to practice. Afterwards, females were housed individually in a standard cage with filtertop and new nesting material, and placed in a ventilated cabinet to provide a standardized and quiet environment. Starting from 18 days after the breeding, females were checked each morning before 09:00 a.m. When a litter was born, the previous day was determined as postnatal day 0.

### 2.2 Early-life stress paradigm

Early-life stress was induced by providing limited nesting and bedding material from postnatal day(P) 2 to P9, as described previously (Naninck et al., 2015; Rice et al., 2008). To avoid differences in maternal care due to litter size, large litters were culled to 6, and no litters of less than 5 pups were included. Control cages had a standard amount of sawdust and one square piece of cotton nesting material (5 × 3x5 cm, Techninlab-BMI, Someren, The Netherlands). ES cages consisted of a little amount of sawdust on the bottom, covered with a fine-gauge stainless steel mesh, and half a square piece of cotton nesting material (2.5 × 3x3 cm). All cages were covered with a filtertop. At P2 and P9 the pups, dams, and food were weighted. At P9, all litters were moved to new cages containing standard amounts of sawdust, and were left undisturbed until weaning at P21.

### 2.3 Bodyweight measurements

Bodyweight (BW) was measured throughout development and diet exposure. At P2, P9, P21, P35 BW was assessed in group-housed animals. BW gain from P2 to P9 was calculated per litter for each sex, by subtracting the average P2 BW from the average P9 BW of all male and female pups respectively. From P63 onwards, animals were individually housed and weighted weekly until the end of the experiment.

### 2.4 Food choice

**Fig. 1:**
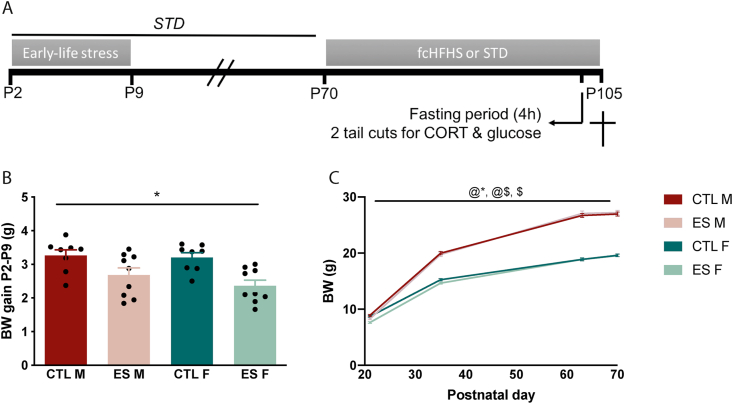
**A)** Experimental overview. Male and female mice were exposed to CTL and ES conditions from P2 to P9, and at 10 weeks of age either fed a standard diet (STD) or free choice high-fat high-sugar choice diet (fcHFHS) for 5 weeks. Body weight and food intake was measured weekly. At the end of the 5th week, blood was taken via a tail cut to measure basal CORT levels. Next, animals were fasted for 4 h and blood was taken again to measure basal glucose levels. Food intake was measured after this manipulation. **B)** BW gain from P2–P9 is lower in ES exposed animals. **C)** BW development from P21 to P70. BW increases with age, and is affected by ES exposure (P21), and sex (P35–P70). Indicated is mean ± SEM, p < 0.05. * main effect of condition; @ main effect of time; $ main effect of sex; @* time by condition interaction effect; @$ time by sex interaction effect.

To allow for adequate food intake measurements, animals were housed individually at 9 weeks of age. After one week of acclimatization, animals were randomly divided into the free choice high-fat high-sugar diet (fcHFHS) group, or control chow group. The control group had *ad libitum* access to regular chow (CRM (P), 801722, Standard Diets Services, Essex, United Kingdom, 3.585 kcal/g, where: 22% protein, 9% fat, and 69% carbohydrates) and tap water. The fcHFHS group had *ad libitum* access to four different components: regular chow; a bottle of tap water; pellets of beef fat (beef tallow, Vandemooretele, France, 9 kcal/g); 10% sugar water (0.4 kcal/mL) for 5 weeks (Fig. 1). For basal measurements, food intake and body weight was measured on a weekly basis. In addition, food intake was measured in the 24 h following a mild stressor composite of the following disturbances: a 4 h fasting period at the start of the light period and two tail cuts (see below). This intrinsic aspect of our experimental design allowed us to assess the effects of such an exposure on food choice. Fasting is considered stressful and increases glucocorticoid levels in mice (Champy et al., 2004). While generally mice tend to eat mostly during the night, after several weeks of HFHS diet exposure, mice eat equal amounts of fat and sugar during the day and night (Blancas-Velazquez et al., 2018), thus the fasting during the light period as performed in our experiment does disrupt food intake patterns of animals on a HFHS diet and thereby can be considered a stressor in combination with the two tail cuts. We measured food intake in the 24 h prior and following this event.

### 2.5 Blood collection and fasting

Blood (30–80 μl) was collected via tail cuts at multiple time points by making a small incision at the base of the tail to measure either corticosterone (CORT) or glucose levels (see Fig. 1A). Blood was drawn (without restraint) within 1 min after removing the mouse from its cage. For plasma CORT measurements, blood was collected between 08:00 and 09:00 a.m. from *ad libitum* fed mice. After this first tail cut, animals were fasted for 4 h to measure blood glucose levels (blood drawn between 12:00 and 01:00 p.m.). In the 24 h following the exposure to this fast and tail cuts, food intake was measured.

### 2.6 Glucose and corticosterone measurements

Blood was collected in EDTA-coated tubes (Sarstedt, The Netherlands), and centrifuged at 13000 rpm for 15 min. Plasma was stored at −40 °C. CORT levels were measured with a radioimmunoassay kit (MP Biomedicals, The Netherlands). Glucose was measured using a FreeStyle Optium Neo meter (Abbott Laboratories, Abbott Park, IL, USA).

### 2.7 Tissue collection

After five weeks of diet exposure, at 15 weeks of age, animals were sacrificed to study the adipose tissue and brain. Mice were anaesthetized by an IP injection of pentobarbital (Euthasol 120 mg/kg), and gonadal white adipose tissue was quickly weighted, snap-frozen, and stored at −80 °C. Afterwards, mice were transcardially perfused with 0.9% saline, followed by 4% paraformaldehyde (PFA) in phosphate buffer (PB, 0.1 M, pH 7.4). Brains were post-fixed in 4% PFA overnight at 4 °C, and stored in PB with 0.01% azide (at 4 °C) until slicing. Brains were cryoprotected with sequentially 15% and 30% sucrose solutions, sliced in 40 μm thick coronal sections, and stored in antifreeze (30% ethylene glycol, 20% glycerol, 50% 0.05 M PBS) at −20 °C.

### 2.8 Real-time PCR

To obtain RNA, adipose tissue was homogenized in TRIzol (Invitrogen, Carlsbad, CA, USA) and samples were centrifuged to remove excessive fat. After the addition of chloroform (Sigma Aldrich, Saint Louis, MO, USA) and more centrifuging, the RNA appeared in the upper (aqueous) phase. Next, a RNA clean and concentrator kit with DNAse I treatment (ZYMO Research, Irvine, CA, USA) was used to obtain clean RNA samples. RNA was stored at −80 °C until cDNA was synthesized with SuperScript II Reverse Transcriptase (Invitrogen, Carlsbad, CA, USA). cDNA was stored at −20 °C until further use. Relative gene expression was assessed by RT-PCR performed on a QuantStudio (TM) 6 Flex System (Thermo Fisher Scientific, Waltham, MA, USA). Hot FirePol EvaGreen Mastermix (Solis Biodyne, Tartu, Estonia), 150 nM of gene specific forward and reverse primers and 0.135 ng/μl cDNA template were added to the reaction mix. Primers (Eurogentec, Liege, Belgium, Table 1) all had an efficiency between 90 and 110%. Cycling conditions were as follows: 15 min polymerase activation at 95 °C and 40 cycles of replication (15 s at 95 °C, 20 s at 65 °C, and 35 s at 72 °C). ΔΔCt method was used to calculate relative gene expression, and was performed in Qbase + software (Biogazelle, Gent, Belgium). Expression was normalized for two reference genes, which were not affected by experimental conditions and tested for stability in Qbase+.

**Table 1:** Primers used for RT-PCR.

Gene	Pathway/function	Forward primer (5′-3′)	Reverse primer (5′-3′)
CD36	Fatty acid uptake	GCAAAGAACAGCAGCAAAATC	CAGTGAAGGCTCAAAGATGG
FABP4	Fatty acid binding protein	GAAATCACCGCAGACGACAG	ATAACACATTCCACCACCAGC
FASN	Fatty acid synthesis	GCGCTCCTCGCTTGTCGTCT	TAGAGCCCAGCCTTCCATCTCCTG
Leptin	Anorexigenic adipokine	AGCTGCAAGGTGCAAGAAGAA	CCTGGACTTTCTGGATAGGCA
PPARγ	Fatty acid storage and glucose metabolism	GTCTCACAATGCCATCAGGTT	CAAATGCTTTGCCAGGGCTC
CANX	Reference gene	AGAGCTCAGCCTGGATCAATTC	TTGTAGTCCTCTCCACACTTATCTG
RPL19	Reference gene	TTGCCTCTAGTGTCCTCCGC	CTTCCTGATCTGCTGACGGG

### 2.9 Fluorescent immunohistochemistry

To study the brain circuits relevant for food intake, brain slices were immuno-stained with agouti-related protein (AgRP), tyrosine hydroxylase (TH), and glutamate decarboxylase 65 (GAD65). First, free-floating brain slices were washed (3 × 10 min) with 0.05 M tris-buffered saline (TBS, pH 7.6). Slices were incubated in 3% bovine serum albumin (BSA) and 0.3% Triton-X100 in 0.05 M TBS (blocking buffer) for 1 h, and subsequently incubated in a primary antibody solution containing goat anti-AgRP (Neuromics, Edina, MN, USA, GT15023, 1:1000), rabbit anti-TH (Pel-Freez, Rogers, AR, USA, P40101-150, 1:1000), and mouse anti-GAD65 (Abcam, Cambridge, UK, 26113, 1:2500) in blocking buffer, for 24 h at room temperature (RT). Following another series of washes in TBS (3 × 10 min), sections were again incubated in blocking buffer for 2 h, followed by an incubation with secondary antibodies overnight at 4 °C (donkey-α-rabbit 488, Invitrogen A21206; donkey-α-goat 568, Invitrogen A11057; donkey-α-mouse 647, Invitrogen A31571, all 1:500, in blocking buffer). Sections were washed and mounted with a DAPI-containing mounting solution. Negative controls containing no primary antibody in the blocking buffer (but a similar treatment otherwise) were taken along to confirm the specificity of the labelling.

### 2.10 Confocal microscopy and analysis

Analysis was done by a researcher blind to experimental conditions. All pictures were taken using a Nikon A1 confocal microscope. For analysis of hypothalamic AgRP, 4 pictures with a 20× objective were taken throughout the arcuate nucleus of the hypothalamus (ARH), between bregma −1.055 and −1.955 (based on Allen Brain Atlas, 2011). For analysis of AgRP in the ventral tegmental area (VTA), also four pictures were obtained with a 20× objective, between bregma −2.48 and −3.68. To obtain a proper representation of the staining throughout each brain section, each picture consisted of a Z-stack with 20 individual photos with a distance of 1 μm, and to achieve a correct representation of the whole brain region (either ARH or VTA), the imaged brain sections had an approximate similar intersection distance. AgRP rapidly diffuses from the cell body into the fibers, and therefore only AgRP fibers were quantified. Fiber density was determined with a thresholding method within a defined region of interest using ImageJ software (National Institutes of Health, Bethesda, MD, USA). First, Z-stacks were collapsed with the sum projection method. Subsequently, a threshold was determined selecting only AgRP positive staining. Finally, for each animal, the obtained area covered by AgRP positive staining was averaged over the different sections, for either the ARH or VTA.

For analysis of TH^+^ cells in the VTA, 4 pictures (Z-stacks) were taken with a 20× objective, similar as described above, and TH^+^ cell bodies were counted in a defined ROI within the VTA. Next, the average number of TH ^+^ cells per μm^2^ over the different pictures was calculated for each animal.

To study presynaptic GABA terminals (GAD65) on dopaminergic cells in the VTA, 4 pictures were taken between bregma −2.88 and −3.38 with a 60× oil objective. Each picture consisted of a Z-stack with 13 individual photo's, with a distance of 1 μm. In these pictures, both GAD65 punctae on TH^+^ cell bodies as well as on TH^+^ fibers were counted. For counting of GAD65 punctae on cell bodies, all cell bodies that were well visible and not touched by other cell bodies were counted (±13 cells per animal) on two different Z planes with 2 μm between them to not count the same punctae double. GAD65 punctae were counted when they were directly touching the cell bodies. The counts of both Z planes of each cell body were added together, and next, an average of all cell bodies was calculated for each animal. To quantify the GAD65 punctae on fibers, two ROIs in each picture were chosen that did contain TH^+^ fibers, but no TH^+^ cell bodies. Similar as for the cell bodies, this was done for 2 different Z planes, with 2 μm between them. In these ROIs, first the area covered by TH^+^ staining was quantified using a set threshold. Next, all punctae touching or directly on top of fibers were counted, and the number of punctae per μm^2^ fiber was calculated for each animal.

### 2.11 Estrous cycle determination

Estrous cycle sampling was performed by gently rubbing an öse along the ventral/rostral side of the vagina directly after a tail cut or when females were anaesthetized (before sacrificing) to minimize extra handling. Vaginal swabs were then transferred to a glass slide (with a drop of water), and stained with Giemsa for 10 min followed by several washed with water. Determination of the estrous cycle stage was done with a light microscope and performed by a trained researcher blinded for experimental conditions.

### 2.12 Statistical analysis

Data were analysed with SPSS 25.0 (IBM software, Armonk, NY, USA), Graphpad Prism 6 (Graphpad software, San Diego, CA, USA) and R Studio 1.2.1335 (R Core Team, 2018). All data are presented as mean ± standard error of the mean (SEM). When p < 0.05, data was considered statistically significant. For statistical analysis of RT-PCR results log transformed values were used. Firstly, outlier analysis was performed in SPSS, and values that were outside the 1st quartile – 3*interquartile range or 3rd quartile +3*interquartile range were excluded. Depending on the measure, this resulted in the exclusion of 0–5 outliers per analysis. For the intake of the different dietary components (chow, fat and sugar), outliers were identified and removed for each individual component (but not for the total caloric intake). If an animal was outlier for one of the components, it was also removed for the total caloric intake measure. For the relative caloric intake of each component (as a percentage of total intake), when an animal was outlier for one of the intakes, that animal was excluded from the analyses of the relative intake of all the other components as well. Because of a priori expectations of sex differences in metabolism as well as in response to ES (Fuente-Martín et al., 2013; Murphy et al., 2017; Naninck et al., 2015), all data were analysed with sex as independent variable. Data with condition (CTL/ES) and sex as predictor variables were analysed with a 2-way or repeated measures 2-way ANOVA. When a 2-way interaction between condition and sex was found, post hoc pairwise comparisons with Bonferroni correction were performed. Data with condition, sex and diet as predicted variables were analysed with a 3-way or repeated measures 3-way ANOVA. When a 3-way interaction between condition, diet and sex was found, the interaction was further explored with simple 2-way interactions, followed by pairwise comparisons with Bonferroni correction. When in a 3-ANOVA, a 2-way interaction was found, because this suggested no contribution of the third predictor to the interaction, we did not explore these 2-way interaction further with pairwise comparisons between each individual experimental group. As multiple mice from one litter were included in these experiments, data are considered nested. We therefore tested for contributing effects of litter and corrected when necessary by performing mixed model analysis with litter as random factor. To test if estrous cycle influenced the outcome variables across the groups including female mice, we tested for contributing effect of estrous cycle and corrected with mixed model analysis with estrous cycle as random factor when necessary, however estrous cycle did not influence any of the outcome variables.

Correlation plots exclusively showing the significant correlations were generated in R studio 1.2.1335 (R Core Team, 2018) for each individual experimental group using the ggcorrplot package. Pearson correlations were calculated based on complete pairwise cases, and correlation coefficients were tested against critical values on a two-tailed distribution (alpha = 0.05).

## 3 Results

### 3.1 Effects of ES on bodyweight

Fig. 1A depicts the experimental overview. Animals were exposed to either CTL or ES conditions between P2 and P9, and fed a STD or fcHFHS from P70 to P105. At P104 animals were exposed to a 4 h period of fasting and 2 tail cuts (mild stress exposure). Bodyweight (BW) was measured throughout the experiment, and during the dietary exposure, food intake was measured on a weekly basis as well as in the 24 h after the mild stress exposure.

ES exposure reduced bodyweight (BW) gain from P2 to P9 in males and females (F_condition_ (1, 30) = 16.291, p < 0.001) (Fig. 1B). In addition, BW development depended on both condition (F_time*condition_ (1.945, 175.075) = 6.973, p = 0.002) and sex (F_sex_ (1, 90) = 532.483, p < 0.001; F_time*sex_ (1.945, 175.075) = 363.617, p < 0.001) (Fig. 1C). Further analysis revealed that at P21, ES reduced BW (F_condition_ (1, 30.335) = 4.404, p = 0.044) independent of sex. However, these ES effects normalized at P35. In addition, males had a higher BW compared to females at P35 (F_sex_ (1, 92) = 350.971, p < 0.001), P63 (F_sex_ (1, 91) = 608.652, p < 0.001), and P70 (F_sex_ (1, 92) = 607.92, p < 0.001).

### 3.2 Males and females have different food choice

**Fig. 2:**
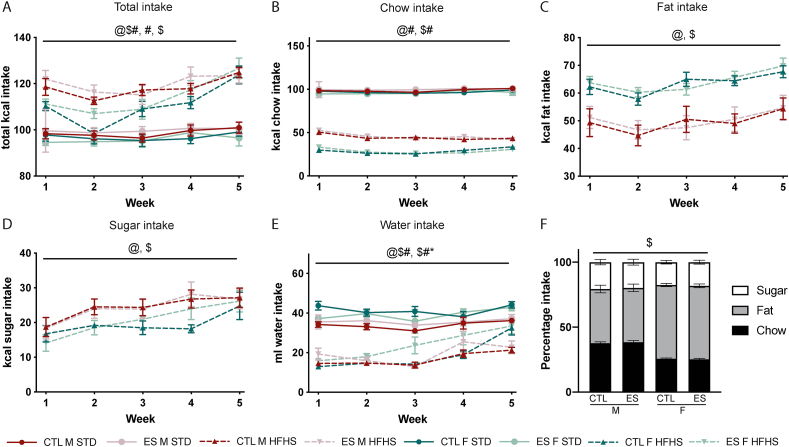
Food intake during the 5-week exposure to either STD or fcHFHS. Shown are kcal intake per week of each component. **A)** total kcal intake per week is affected by diet and sex. **B)** kcal intake from chow per week is affected by diet and sex. C) Kcal fat intake is higher in females. D) Kcal intake from sugar is higher in males. **E)** Water intake per week is higher affected by sex and diet**. F)** The relative intake of chow and fat as a percentage of total caloric intake is affected by sex. Indicated is mean ± SEM, p < 0.05. @ main effect of time; # main effect of diet; $ main effect of sex; @# time by diet interaction effect; @$ time by sex interaction effect; #$ diet by sex interaction effect; @$# time by sex by diet interaction effect.

To investigate whether ES and sex affect caloric intake and/or food choice, food intake was measured in CTL and ES-exposed male and female mice on a weekly basis during the 5-week dietary exposure. Food intake was affected in the following manner: *total* caloric intake was affected by time, diet and sex (F_sex_ (1, 66) = 6.999, p = 0.01; F_diet_ (1, 66) = 84.572, p < 0.001; F_time*diet*sex_ (3.032, 200.132) = 2.734, p < 0.001). Mice on STD had lower caloric intake compared to animals on fcHFHS, and fcHFHS fed mice exhibited increased intake over time with males having a higher intake compared to females (Fig. 2A). Kcal *chow* intake over the 5-week period was higher in STD versus fcHFHS fed animals independent of sex (F_diet_ (1, 77) = 2466.161, p < 0.001; F_time*diet_ (3.022, 232.693) = 4.456, p = 0.004) (Fig. 2B). Moreover, across the fcHFHS fed groups, males ate more chow compared to females (F_sex*diet_ (1, 77) = 27.507, p < 0.001). Interestingly, while in both sexes, *fat* and *sugar* intake increased over time (fat: F_time_ (2.606, 130.295) = 10.544, p < 0.001; sugar: F_time_ (2.629, 107.728) = 20.774, p < 0.001), females had more kcal intake from fat compared to males (F_sex_ (1, 50) = 23.167, p < 0.001) (Fig. 2C), while males had a higher kcal sugar intake compared to females (F_sex_ (1, 41) = 5.099, p = 0.029) (Fig. 2D). ES exposure did not affect chow, fat or sugar intake over the 5-week period in either sex. Water intake over the 5 week period was affected by time, sex, diet and condition (F_time*diet*sex_ (3.023, 220.678) = 3.333, p = 0.02; F_condition*sex*diet_ (1, 73) = 5.235, p = 0.025) (Fig. 2E). Water intake was higher in animals on STD diet compared to those on HFHS diet, and females drank more water compared to males, but this depended on ES exposure and diet.

In addition, we analysed the *percentage* kcal intake for each diet component based on their average total intake over the 5-week period, specifically for the animals exposed to the fcHFHS (Fig. 2F). Males took relatively more kcal from chow compared to females (F_sex_ (1, 51) = 187.234, p < 0.001), while females took relatively more kcal from fat compared to males (F_sex_ (1, 51) = 42.647, p < 0.001) (Fig. 2F). ES did not modulate choice for any of the components.

### 3.3 ES-exposed females increase fat intake after a mild stressor (4 h fast and 2 tail cuts)

**Fig. 3:**
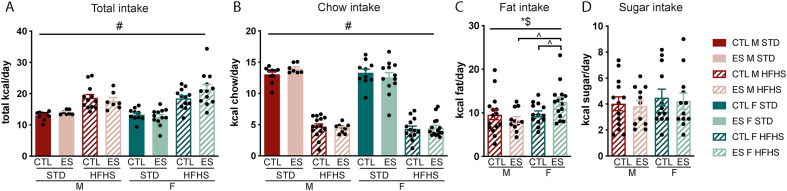
Food intake after mild stress exposure **A)** Total caloric intake is higher in animals exposed to fcHFHS. **B)** kcal chow intake is lower in the fcHFHS groups. **C)** Kcal fat intake is higher in ES exposed females. **D)** Kcal sugar intake is not affected by sex or condition. Indicated is mean ± SEM, p < 0.05. # main effect of diet; $ main effect of sex; *$ condition by sex interaction effect; ^∧^ significant Bonferroni post hoc comparison between experimental groups.

Palatable foods are considered comforting, and acute stress exposure alters the intake of such foods (Dallman et al., 2004; Rutters et al., 2009). Our experimental design offered the opportunity to test the effects of a mild stressor in the form of a 4 h fast and 2 tail cuts during the 5th week of dietary exposure on food choice. First, as a baseline measurement, we analysed caloric intake in the 24 h directly *before* the mild stressor (Fig. S1). In the 24 h prior to the mild stressor, total kcal intake per day was higher in animals exposed to the fcHFHS (F_diet_ (1, 78) = 21.153, p < 0.001), and not affected by condition or sex (Fig. S1A). Kcal chow intake per day was lower in the fcHFHS groups, and lower in females when compared to males (F_diet_ (1, 86) = 498.061, p < 0.001; F_sex_ (1, 86) = 7.49, p = 0.008) (Fig. S1B). Fat intake was, as expected, higher in females but not affected by ES exposure (F_sex_ (1, 50) = 4.234, p = 0.045) (Fig. S1C). Kcal sugar intake per 24 h was not affected by sex or condition (Fig. S1D). Thus, ES exposure did not affect intake of any of the components, nor total intake, before the exposure to this mild stress. Subsequently, we analysed the caloric intake of the different components in the 24 h *after* the mild stressor. Total caloric intake was higher in the fcHFHS groups (F_diet_ (1, 82) = 63.826, p=<0.001) (Fig. 3A), while chow intake was lower in the fcHFHS groups (F_diet_ (1, 83) = 567.452, p < 0.001) (Fig. 3B). Both total and chow intake were unaffected by condition or sex or the interaction of these predictor variables. Fat intake was affected by sex depending on previous ES exposure (F_sex*condition_ (1, 50) = 4.043, p = 0.05) (Fig. 3C). Post hoc analysis revealed that specifically ES-exposed females showed increased fat intake compared to CTL females (p = 0.049), whereas no such ES effects were observed in males (p = 0.39). Moreover, ES females ate more fat than ES males (p = 0.005). Sugar intake was not affected by sex or ES exposure (Fig. 3D).

### 3.4 ES exposure affects bodyweight gain and adiposity in a sex-dependent manner

**Fig. 4:**
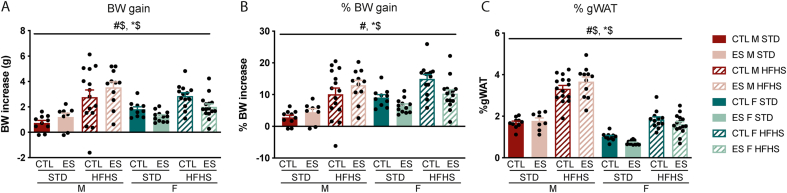
Effects of ES and fcHFHS on BW gain and gWAT in male and female mice**. A)** BW gain is affected by diet, ES exposure and sex. **B)** gWAT is affected by diet, ES exposure and sex. Indicated is mean ± SEM, p < 0.05. # diet effect; $ sex effect; #$ diet by sex interaction effect; *$ condition by sex interaction effect.

Next, we investigated the effects of STD and fcHFHS on BW gain and adiposity (Fig. 4). After 5 weeks of diet exposure, BW gain was higher in fcHFHS fed animals compared to STD fed animals, and while BW gain on STD was higher in females compared to males, BW gain on fcHFHS was higher in males than in females (F_diet*sex_ (1, 86) = 6.66, p = 0.012) (Fig. 4A). ES exposure also affected BW gain depending on sex, so that females exposed to ES had lower BW gain compared to CTL females, while this was not the case in males (F_condition*sex_ (1, 86) = 6.931, p = 0.01). When adjusting for sex differences in body size by taking the BW gain as a percentage of their BW at the start of diet exposure, it was shown that the fcHFHS increased %BW gain (F_diet_ (1, 86) = 40.138, p < 0.001), and that ES reduced the %BW gain in females independent of diet (F_condition*sex_ (1, 86) = 8.538, p = 0.004).

gWAT levels (as percentage of BW) were lower in females compared to males, but increased in fcHFHS fed mice in both sexes (F_sex*diet_ (1, 84) = 21.563, p < 0.001) (Fig. 4C). In addition, there was an interaction between ES exposure and sex in their effect on gWAT levels (F_condition*sex_ (1, 84) = 6.121, p = 0.015) indicating that ES decreased gWAT levels in females.

### 3.5 The physiological response to the fcHFHS is dependent on sex

**Fig. 5:**
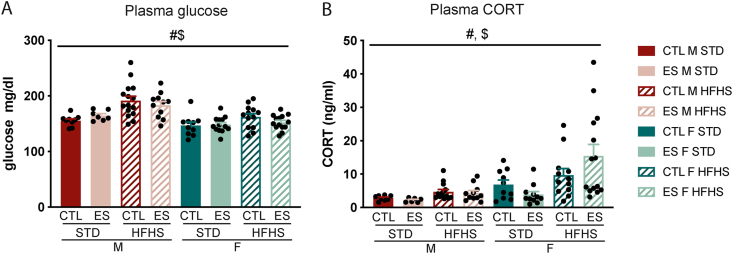
Effects of ES and fcHFHS on circulating glucose and hormones in males and females. **A)** Circulating glucose levels are higher in fcHFHS exposed animals depending on sex. **D)** CORT levels are higher in animals exposed to fcHFHS and higher in females. Indicated is mean ± SEM, p < 0.05. # diet effect; $ sex effect; #$ diet by sex interaction effect.

A 5-week exposure to the fcHFHS increased circulating glucose levels in males, but not females (F_diet*sex_ (1, 84) = 4.265, p = 0.042) (Fig. 5A). CORT levels were higher in animals fed fcHFHS (F_diet_ (1, 77) = 10.309, p = 0.002), and higher in females compared to males (F_sex_ (1, 77) = 15.278, p < 0.001) (Fig. 5B).

### 3.6 Adipose tissue metabolism-related gene expression is affected by ES exposure and sex

**Fig. 6:**
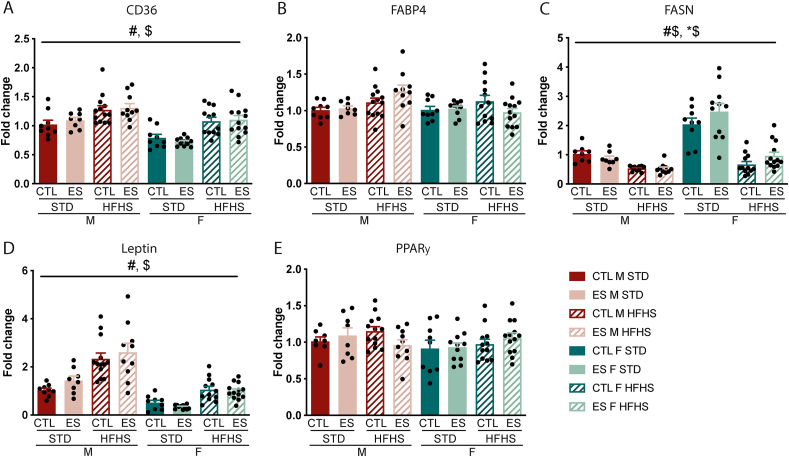
Effects of ES exposure and diet on adipose tissue gene expression in males and females. **A)** CD36 expression is higher in fcHFHS exposed animals and lower in females. **B)** ES, diet and sex do not affect FABP4 expression. **C)** FASN expression is affected by diet, sex and condition. **D)** Leptin expression is higher in males and in animals exposed to the fcHFHS **E)** PPARγ expression is not affected by diet, condition or sex. Indicated is mean ± SEM, p < 0.05. # main effect of diet; $ main effect of sex; #$ diet by sex interaction effect; *$ condition by sex interaction effect.

To further understand the effects of ES and sex on the adipose tissue after both STD and fcHFHS exposure, we studied the expression of genes involved in fatty acid metabolism and the adipokine leptin in the gonadal adipose tissue. Expression of CD36, a gene involved in fatty acid uptake, was elevated in animals on fcHFHS compared to those on STD (F_diet_ (1, 77) = 42.85, p < 0.001), and lower in females compared to males (F_sex_ (1, 77) = 36.281, p < 0.001) (Fig. 6A). FABP4, a fatty acid binding protein, was not affected by any of the predictor variables (Fig. 6B). Expression of FASN, involved in fatty acid synthesis, was higher in females fed STD (F_diet*sex_ (1, 75) = 8.91, p = 0.004) (Fig. 6C). In addition, ES exposure and sex interacted in their effect on FASN expression (F_condition*sex_ (1, 75) = 5.54, p = 0.021): ES increased FASN expression in females but not males. The gene expression of the adipokine leptin was higher in animals exposed to the fcHFHS (F_diet_ (1, 77) = 71.508, p < 0.001) and higher in males compared to females (F_sex_ (1, 77) = 114.327, p < 0.001) (Fig. 6D). Finally, PPARγ expression, involved in fatty acid storage and glucose metabolism, was unaffected by any of the predictor variables (Fig. 6E).

### 3.7 ES affects the brain circuits regulating food intake in a sex-dependent manner

**Fig. 7:**
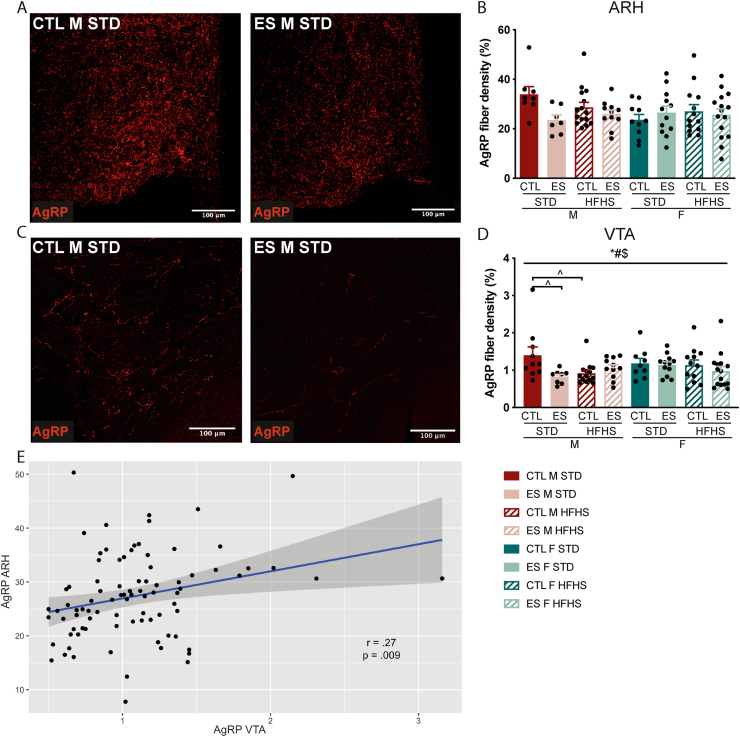
**A)** Representative pictures of AgRP staining in the ARH **B)** AgRP fiber density in the ARH was not affected by ES exposure or diet in males and females. **C)** Representative images of AgRP staining in the VTA. **D)** AgRP in the VTA was affected by the interaction between condition, diet and sex. **E)** AgRP in the ARH and VTA correlate. Indicated is mean ± SEM, p < 0.05. *#$ condition by diet by sex interaction effect; ^∧^ significant Bonferroni post hoc comparison between experimental groups.

We next investigated if the brain circuits that regulate food intake are affected by ES, sex and/or diet. In the ARH, ES, diet, and sex did not affect AgRP fiber density (Fig. 7A and B). In the VTA however, AgRP fiber density was affected depending on ES exposure, diet and sex (F_condition*diet*sex_ (1, 83) = 5.434, p = 0.022) (Fig. 7C and D). When further exploring this 3-way interaction by stratifying on sex, we observed a condition by diet interaction effect in males (F (1, 83) = 7.547, p = 0.007), but not females. Pairwise comparisons between the male groups showed that on STD, ES males had lower AgRP fiber density in the VTA compared to CTL males (p = 0.004). In addition, the fcHFHS lowered AgRP fiber density in CTL males (p = 0.006) to a similar level as ES males on fcHFHS (p = 0.407). Finally, even though AgRP in the ARH was not significantly affected by any of the predictor variables, AgRP in the ARH and VTA did correlate (r = .27, p = 0.009) (Fig. 7E).

**Fig. 8:**
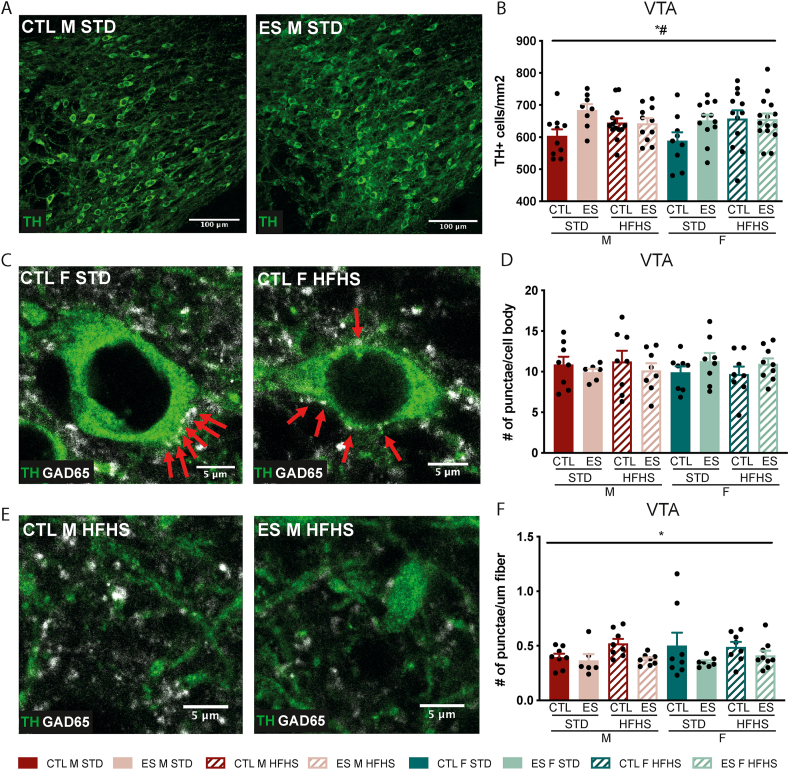
Effects of ES and fcHFHS on TH + cells and inhibitory input in the VTA. **A)** Representative images of TH + staining in the VTA. **B)** ES exposure affects TH + cell numbers depending on diet. **C)** Representative images of GAD65 punctea on TH ^+^ cell bodies. The red arrows indicate counted GAD65 punctae. **D)** GAD65 punctae on TH^+^ cell bodies was not affected by condition, diet or sex. **E)** Representative images of GAD65 punctae on TH + fibers. **F)** GAD65 punctae on TH + fibers was lower in ES-exposed animals. Indicated is mean ± SEM, p < 0.05. * main effect of condition; *# condition by sex interaction effect. (For interpretation of the references to colour in this figure legend, the reader is referred to the Web version of this article.)

Next, we investigated the number of dopaminergic cells as indicated by TH^+^ cell bodies in the VTA. TH^+^ cell density was higher in ES exposed animals compared to CTL animals, but only when fed STD (F_condition*diet_ (1, 83) = 6.527, p = 0.012) (Fig. 8A and B). Moreover, we investigated the number of inhibitory pre-synapses indicated by GAD65 punctae on TH^+^ fibers and TH^+^ cell bodies. There were no effects of ES, diet or sex on GAD65 punctae on cell bodies (Fig. 8C and D). However, ES decreased the number of punctae on fibers (F_condition_ (1, 54) = 6.315, p = 0.015), with no further modulation by diet or sex (Fig. 8E and F).

### 3.8 Correlations between food intake, brain parameters and physiological response to the fcHFHS

**Fig. 9:**
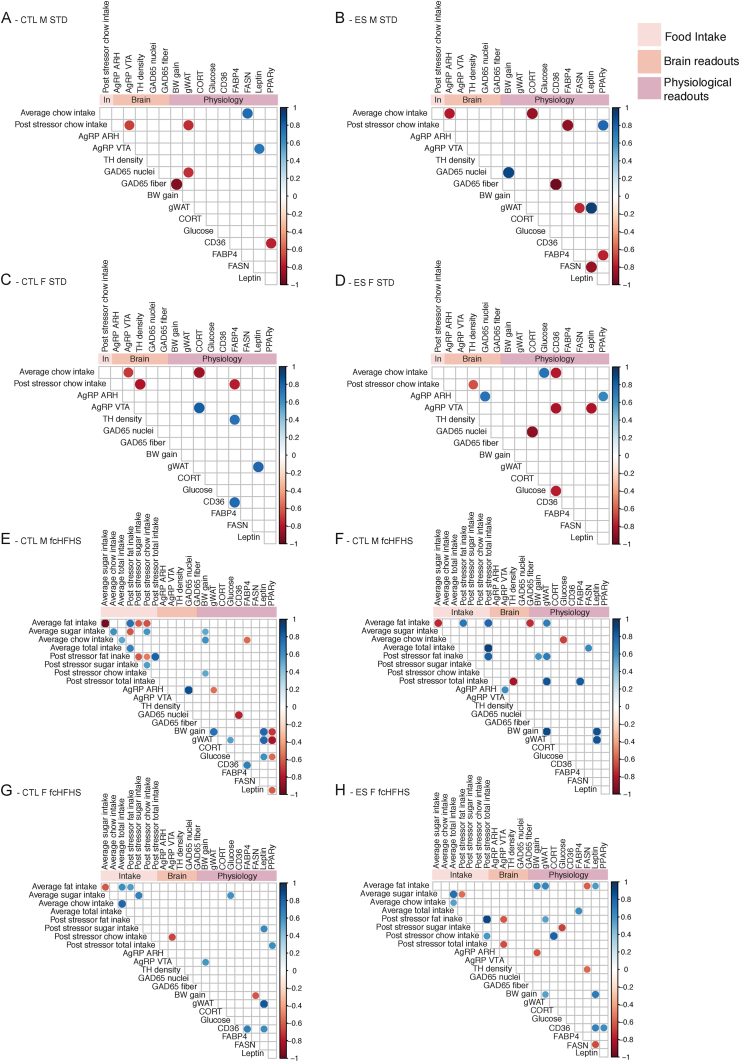
Correlation plots showing significant correlations in CTL M STD (A), ES M STD (B), CTL F STD (C), ES F STD (D), CTL M fcHFHS (E), ES M fcHFHS (F), CTL F fcHFHS (G) and ES F fcHFHS (H). Blue indicates a positive correlation, red indicates a negative correlation, and the larger/darker the dot the stronger the correlation. (For interpretation of the references to colour in this figure legend, the reader is referred to the Web version of this article.)

Our experimental design enables us to study the correlations between the assessed parameters across the experimental groups in more detail. This may give insights in how the regulation of food intake and metabolism is affected by early-life condition, sex and diet. Fig. 9 displays the correlation plots for each experimental group showing only the significant correlations: CTL M STD (Fig. 9A), ES M STD (Fig. 9B), CTL F STD (Fig. 9C), ES F STD (Fig. 9D), CTL M HFHS (Fig. 9E), ES M HFHS (Fig. 9F), CTL F HFHS (Fig. 9G), and ES F HFHS (Fig. 9H). The different experimental groups show different correlation patterns between food intake, brain and metabolic measures.

## 4 Discussion

**Fig. 10:**
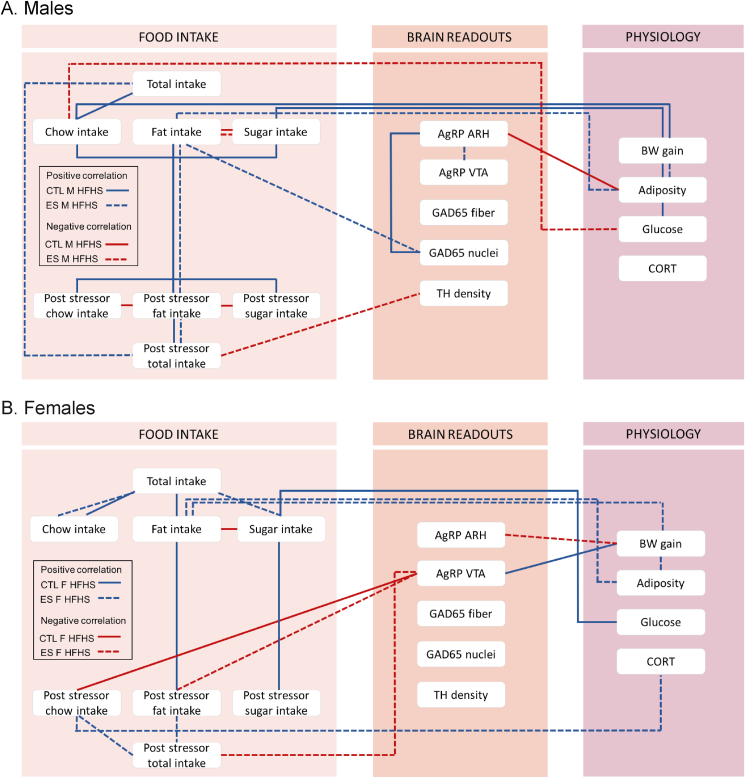
Representation of some of the salient correlations in the groups exposed to the fcHFHS in CTL and ES males (A), and CTL and ES females (B).

We investigated if and how ES exposure affects food choice on a fcHFHS, the physiological response to this diet, the feeding-related neuronal circuits, and if sex impacts this. We here show for the first time that i) males compared to females choose to eat more chow and sugar while females eat more fat. ii) ES does not modulate total intake and food choice for any of the components under basal state, however, after exposure to a stressful event (in the form of 4 h a fasting and 2 tail cuts) specifically ES-exposed females increased their fat intake. Moreover, iii) while as one would expect, the 5-week fcHFHS exposure increased BW gain across groups, ES-exposed females showed lower BW gain compared to CTL females with no such effects in males. iv) Expression of genes important for lipid metabolism in the adipose tissue were affected by the diet in a sex dependent manner. v) Finally, the brain circuits that regulate (palatable) food intake (encompassing AgRP, TH and the inhibitory inputs modulating TH in the VTA) were affected by both ES and diet exposure, partly in a sex-dependent manner. When taking all the data together and exploring the correlations between the various aspects, an interesting overall sexually dimorphic picture emerges (Figs. 9 and 10). Below we will discuss our findings in more detail, starting with the sex differences in food choice, followed by sex-specific modulations by ES on food choice. Next, sex differences in the physiological response to the diet will be discussed, as well as sex-specific effects of ES on these parameters. We will finish by deliberating on the neural circuits that could be involved, and how sex and ES impact these.

### 4.1 Sex impacts on food choice

We observed a clear sex difference in food choice with males collecting a more carbohydrate-rich diet (chow and sugar) compared to females, and females choosing a more fat-rich diet. To the best of our knowledge, we are the first to address this question with our fcHFHS paradigm, unique in offering the choice between 4 separate components: standard chow, water, fat and sugar water. In fact, very few pre-clinical studies investigated sex differences in food choice, and none with our paradigm. Several studies investigated the effect of physical exercise on food choice in males and females, and showed that running induced avoidance of high-fat diet (HFD) only in males (Lee et al., 2017), or for a longer time period in males compared to females (Yang et al, 2019, 2020). In the sedentary controls in these studies, and in contrast to our findings, there was no sex difference in HFD preference (Yang et al., 2019). However, this diet also contained higher sucrose levels and sedentary control cages were enriched with a locked running wheel, thus a direct comparison of the findings is difficult. In addition, human studies also show sex differences in food intake and choice. For example, women have greater trust in healthy nutrition, and a higher fruit and vegetable intake, while men prefer fatty meals with a strong taste and fat-rich meat (Beardsworth et al., 2002; Grzymisławska et al., 2020; Spinelli et al., 2020). However, food choice in humans is confounded by sociocultural and psychological factors (Grzymisławska et al., 2020). Thus, more research is needed to further increase our understanding in sex differences in food choice.

### 4.2 Effects of ES on food choice are sex dependent

We did not observe ES effects on food choice under basal circumstances, but importantly, after exposure to a mild acute stressor, ES-exposed females ate more fat, whereas sugar and chow intake were not affected. Previous studies addressing effects of ES on food choice have used different forms of palatable food and/or have not addressed this question in both sexes, making direct comparisons with our data difficult. However, Machado and colleagues showed that female rats exposed to the limited nesting and bedding (LBN) model had increased preference for the palatable food (combined high-fat high-sucrose pellet) during a 4 week choice paradigm, which they did not test in males (Machado et al., 2013). A recent study found that maternal separation (MS) in rats increased the intake of palatable food (consisting of condensed milk, sugar and milk powder) in both males and females during a 7 day exposure in adulthood, whereas exposure to maternal deprivation (MD) in rats had opposite effects in the two sexes (de Lima et al., 2020). In contrast, other studies showed that ES induced by repeated cross fostering or MS followed by social isolation decreased palatable food (chocolate) induced place preference in female mice, and that LBN decreased chocolate intake in male rats (not tested in females) (Bolton et al., 2018b; Sasagawa et al., 2017; Ventura et al., 2013). Moreover, studies in which the choice was given between normal water and sucrose water, next to their standard chow, have shown that ES either reduced sucrose preference (Bolton et al., 2018a), increased sucrose intake but only in males (Michaels and Holtzman, 2007), or had no effect when exposed to a sucrose water option for a prolonged time (12 weeks) (Maniam et al., 2015). Thus, rodent studies show mixed results on the effects of ES on food choice. Diet composition (e.g. combined palatable chow versus multiple component choice diet) and form (solid versus liquid) affect food intake behaviour and should be taken into account in future studies (la Fleur et al., 2010; La Fleur et al., 2014). Importantly, human studies suggest that ES affects food preference (Jackson and Vaughn, 2019; Lussana et al., 2008), which however could be confounded by environmental factors (more exposure to unhealthy foods) and later life stress exposure.

We specifically observed increased fat intake in ES-exposed females after acute stress exposure. Indeed, palatable foods are considered comforting and affect the HPA-axis (Dallman et al., 2004): stress exposure increases the intake of comfort foods, which in turn can reduce the HPA-axis response to stress (Pecoraro et al., 2004), and CORT increases the intake of fat but not chow in a dose-response manner (La Fleur et al., 2004). In line, ES exposure increased anxiety-like behaviour in rats, which could be reduced by HFHS feeding (combined pellet with 43% fat and 40% sucrose) (Maniam et al., 2016), and high CORT responsive women eat more comfort foods compared to low responders (Epel et al., 2001) (not investigated in men). Indeed, women might be more prone to comfort feeding: when exposed to a stressful task, women increased chocolate candy intake as compared to women exposed to a non-stressful task, whereas men decreased their snack intake (Zellner et al, 2006, 2007). Our data are thus in line with such increased vulnerability in women/females.

### 4.3 Sex impacts the physiological response to fcHFHS exposure

Next to a strong sex effect in food choice, we also found a strong sex difference in the physiological responses to the fcHFHS: males had higher gWAT levels upon fcHFHS exposure compared to females, and only males showed fcHFHS-induced increased glucose levels. Increased glucose levels after 4 weeks of fcHFHS has been described before in male rats accompanied by increased insulin levels and decreased glucose tolerance (La Fleur et al., 2011). We now show a similar effect on glucose levels in male, but not female mice after 5 weeks of fcHFHS. Although this sexual dimorphic response to the fcHFHS could partly be mediated by their altered food choice and thus intake, such sex differences in the response to non-choice high-caloric diets have been described before. Compared to females, males fed a HFD have been shown to display higher weight gain, higher fat mass indexes, and more greatly impaired glucose tolerance (Estrany et al, 2011, 2013; Garg et al., 2011; Grove et al., 2010). Both the quantity and functioning of fat depots are different for males and females (Fuente-Martín et al., 2013; Power and Schulkin, 2008). Different fat depots vary in their adipokine production, free fatty acid release, inflammatory response and development of insulin sensitivity in a sex-specific manner (Macotela et al., 2009; Power and Schulkin, 2008; Wajchenberg et al., 2000). These differences in fat depot functioning, together with the fact that fat distribution is different between men and women, thus has functional implications. Indeed, we observed sex differences in adipose tissue gene expression. CD36 and leptin expression, involved in fatty acid uptake and satiety respectively, were higher in males on both STD and fcHFHS, whereas females on STD had higher FASN (fatty acid synthase) expression. Sex differences in gene expression in the different adipose depots have been described before (Estrany et al., 2013; Grove et al., 2010). For example, males responded to a 12-week HFD exposure by upregulating inflammatory gene expression, while females were shown to have higher expression of genes related to insulin signaling and lipid synthesis, independent of diet (Grove et al., 2010). The sympathetic innervation, as well as the projection from the hypothalamus to WAT differ between males and females (Adler et al., 2012) and may underlie some of these differences in metabolic vulnerability between the sexes.

Finally, we found that 5 weeks of fcHFHS increased basal CORT levels in both males and females, and that females had remarkably higher CORT levels than males. Indeed, while comfort foods may reduce the stress response on the short-term (Pecoraro et al., 2004), long-term HFD feeding as well as obesity have been related to increased circulating CORT levels (Cano et al., 2008; van Rossum, 2017), and females have before been shown to have higher CORT levels compared to males in mice (Naninck et al., 2015). CORT is known to increase blood glucose levels (Kuo et al., 2015), however, while females had higher CORT levels compared to males, we observed an opposite sex effect on glucose levels with females having lower glucose levels and being protected from the HFHS-induced increase in glucose. This suggests other factors, such as the above mentioned adipose distribution and quantity, as well as sex hormones (Varlamov et al., 2014) might contribute to higher glucose levels in males.

### 4.4 The effect of ES on physiological readouts depends on diet and sex

ES exposed females had lower BW gain and gWAT levels compared to controls both on STD and fcHFHS, while for males no such ES effect was observed, and if any, ES seemed to increase BW gain in males. Previous studies showed that on standard chow diet, ES affected adiposity similarly in both sexes, although the directionality depends on the used ES model. For example, ES induced by MS has been found to be increase adiposity in both sexes (Jaimes-Hoy et al., 2016; Murphy et al., 2018), whereas LBN is shown to decrease adiposity in males and females (Yam et al., 2017a). In contrast, when fed a western-style diet with 39.8% fat content for 8 weeks, LBN exposure led to increased fat accumulation in males compared to controls (not performed in females) (Yam et al., 2017a). Studies using MS as ES paradigm did not observe effects of ES when males were fed a HFD, while female adiposity was increased upon 12 and 16 weeks of HFD respectively (60% fat) (Murphy et al, 2017, 2018). Differences in the used ES model as well as differences in diet composition and duration could contribute to these discrepancies. In our study, the lower BW gain and gWAT levels in ES-exposed females could not be explained by lower food intake, raising the question whether ES increases energy expenditure in females. Literature on the effect of ES on energy expenditure is sparse. We have previously shown that ES increases the expression of a gene critical in heat production in both sexes at P9, but not in adulthood (Yam et al., 2017a). ES has also been reported to affect locomotor activity levels (Aya-Ramos et al., 2017; Hancock and Grant, 2009), as well as the hypothalamic-pituitary-thyroid axis (in both sexes), which is key in regulating metabolic rate (Jaimes-Hoy et al., 2019a; Mullur et al., 2014). However, to the best of our knowledge no studies measured effects of ES on metabolic rate itself. It thus remains to be determined what leads to the lower BW gain and gWAT levels in ES-exposed female offspring, and whether there is a role for energy expenditure.

Besides lower adiposity, we also observed higher adipose tissue FASN expression in ES-exposed females. Higher FASN expression has been observed in normoglycemic versus hyperglycemic individuals (Mayas et al., 2010), and dexamethasone (CORT analogue) increases adipose FASN expression *in vivo* (Wang et al., 2004). A higher FASN expression in females is thus in line with the observed lower glucose and higher CORT in females compared to males, but cannot explain the increased FASN in ES females. Interestingly, while in CTL fcHFHS fed females FASN was negatively associated with BW gain, in ES females on fcHFHS, FASN negatively correlated with fat intake but not with physiological measures such as BW gain or adiposity, suggesting FASN expression might be more sensitive to food intake in ES compared to CTL females.

We did not find effects of ES on glucose levels in both males and females, nor in interaction with the diet. Hyperglycemia is a sign of impaired insulin sensitivity, and previous studies have found ES to either have no effect, increase or decrease (various measures of) insulin sensitivity, partly depending on diet and sex of the animal, with males potentially being more vulnerable to develop insulin insensitivity (Jaimes-Hoy et al., 2019b; Maniam and Morris, 2010; Mela et al., 2012; Murphy et al., 2017). To better understand if ES affects insulin sensitivity it would be important to perform, next to basal glucose and insulin measurements, insulin and glucose tolerance tests.

In addition, we did not observe ES effects on adult basal CORT levels under any of the dietary exposures, in line with Naninck et al. (2015) (Naninck et al., 2015). Other studies did show effects of ES on basal CORT levels (Rice et al., 2008), as well as in response to an acute stressor or upon 12 weeks of HFD feeding (Machado et al., 2013; Murphy et al., 2017). As these studies were performed in rats, the possibility of interspecies differences should be kept in mind. Nonetheless, they also indicate that latent effects of ES on CORT could potentially be unmasked upon an acute challenge or more severe/prolonged HFD.

### 4.5 ES affects the brain circuits involved in hedonic driven food intake

Similar as previously reported, the fcHFHS did not affect *hypothalamic* AgRP levels, involved in homeostatic-driven food intake (la Fleur et al., 2010), nor was it different between males and females or affected by ES. However, we did find that AgRP in the *VTA* was decreased in ES-exposed males on STD compared to their respective controls, and that the fcHFHS also reduced AgRP in CTL males. In contrast, in females no such effects of ES or diet were observed. Studies focusing on AgRP in the VTA are rare, and to the best of our knowledge we are the first to describe modulations of ES, diet and sex on VTA AgRP. It has been shown that hypothalamic AgRP neurons innervate the VTA and determine the reward circuit setpoint by affecting dopamine cell excitability and dopamine levels (Dietrich et al., 2012). Notably, ablating hypothalamic AgRP (thereby also ablating the AgRP projections to the VTA) increases palatable food intake, and has been proposed as a model for comfort feeding (Denis et al., 2015). Despite the absence of a significant effect of ES and/or sex on ARH AgRP, AgRP fiber density in VTA and ARH positively correlated, which might suggest that these alterations in the VTA might partly derive from the ARH. However, additional studies are needed to further understand the origin of the changes in the VTA. Moreover, some interesting sex dependent effects are worth noting. For example, despite a reduction in VTA AgRP specifically in males by ES and diet, we did not observe effects of ES on food choice in males, nor did AgRP levels in the VTA correlate with intake parameters. In contrast, in females, where none of the conditions impacted on VTA AgRP, there was a negative correlation between VTA AgRP and fat intake after acute stress specifically in ES-exposed females, while in CTL females VTA AgRP correlated negatively to chow intake after acute stress. The emerging picture seems thus to be that the sex-dependent modulation of AgRP by the various conditions (i.e. ES and diet) might be related to the differential food intake exhibited by males and females after a stress exposure, although this hypothesis needs further exploration.

We observed increased TH^+^ cell numbers (indication of dopamine (DA) cell density) in the VTA of ES-exposed males and females, when fed STD but not when fed fcHFHS. Moreover, independent of diet exposure, we report decreased inhibitory input on TH^+^ fibers in ES-exposed males and females. The mesocorticolimbic DA circuitry originates from VTA DA neurons that connect to limbic structures and the medial prefrontal cortex. This VTA network senses and links both the internal state and the appraisal of environmental stimuli, and in that way establishes emotional-motivational valuations (Douma and de Kloet, 2020). Palatable foods activate the reward circuitry (De Macedo et al., 2016) and both acute and chronic stress have been shown to affect DA release and DA neuron firing (Belujon and Grace, 2015; Chang and Grace, 2014; Holly and Miczek, 2016; Kaufling, 2019; Rincón-Cortés and Grace, 2017). The dopaminergic reward system continues to mature into adolescence (Kalsbeek et al., 1988; McCutcheon et al., 2012; Teicher et al., 1995; Voorn et al., 1988), and stress during this developmental period can affect its functioning later in life. In fact, ES alters multiple aspects of the reward circuitry. For example, ES exposure increased excitability of putative DA neurons in the VTA (Spyrka et al., 2020), blunted DA outflow in the prefrontal cortex (Ventura et al., 2013), lowered DA transporter sites in the striatum and NAcc (Brake et al., 2004), led to transcriptional changes in the VTA (Peña et al., 2017), and increased neuronal activity in the NAcc core (Bolton et al., 2018a). As most studies only included either males or females, it is unclear whether ES alters reward circuitry functioning in a sexually dimorphic manner.

In our study, the fcHFHS seems to overrule the more subtle ES effects on TH^+^ cell numbers, although the ES effect on inhibitory input (GABAergic synapses) on TH^+^ fibers remains. Approximately 30% of cells in the VTA are GABA neurons (the majority of non-DA cells in VTA) (Bouarab et al., 2019), which provide both local and long-range inhibition. The VTA GABA neurons synapse mostly on the proximal dendrites of DA neurons, while (inhibitory) inputs from other regions including the ventral pallidum and laterodorsal tegmentum synapse onto the cell body (Omelchenico and Sesack, 2005; Omelchenko and Sesack, 2009). Of note, these GABAergic VTA neurons are affected by stress, and (through their inhibition of DA neurons) also regulate reward. Acute stressors directly increase the firing rate of VTA GABA neurons thereby suppressing DA firing (Cohen et al., 2012; Tan et al., 2012), while over the days following the stress GABAergic plasticity is lost, removing VTA DA neuron inhibition (Niehaus et al., 2010; Polter et al, 2014, 2017). Although speculative, it is possible that the reduction in GABAergic input on TH^+^ fibers in ES animals is at least partly derived from a reduction in VTA GABA input onto these neurons. An ES-induced reduction of inhibitory input on TH^+^ fibers is in line with the previously described higher excitability of DA neurons (Spyrka et al., 2020). It however is important to note that the VTA also receives input from adrenergic and noradrenergic neurons located in e.g. the caudal medulla and locus ceruleus, which express TH as well (Mejías-Aponte et al., 2009). Therefore DA producing neurons will not be the only origin of the TH^+^ fibers in the VTA.

Importantly, VTA DA neurons have been proposed to function differently between males and females. For example, females have higher VTA DA turnover and release (Becker and Chartoff, 2019) and the afferents to the VTA are also sexually dimorphic (Bale and Epperson, 2015). Moreover, CORT (which in our study was higher in females) directly affects the reward circuitry: adrenalectomy decreases DA release and DA transporters binding in the NAcc shell (Sarnyai et al., 1998). We did not observe sex differences in DA cell density or inhibitory input on DA neurons, but AgRP input in the VTA was affected by ES specifically in males, while it correlated with acute-stress induced food intake only in females. Thus, the circuits that regulate comfort feeding are sexually dimorphic, and besides the fact that ES impacts on aspects of this circuitry in both males and females, ES also has some sex-dependent effects. Sex differences in reward system functioning, together with sex differences in CORT, could be involved in the different vulnerability to comfort feeding between males and females.

### 4.6 General conclusion

We show, for the first time, that males and females make different food choices, and that ES exposure affects food choice after a mild acute stressful event only in females. Moreover, physiologically, males and females respond differently to the diet as well as to ES exposure. Finally, we provide evidence that ES alters the reward circuitry of both male and female mice, partly in a sex-specific manner. Although it remains to be understood how the different elements interact, a different picture emerges in males and females depending on previous ES exposure (Fig. 10). This suggests not only that food choice and metabolism are differently regulated between sexes, but also that ES has a different impact on males and females. Our data highlights the importance of including both sexes in future studies.

## Funding

AK is supported by 10.13039/501100003246NWO Meerwoud, NWO Food cognition and Behaviors and JPI NutriCog.

## CRediT authorship contribution statement

**S.R. Ruigrok:** Conceptualization, Formal analysis, Investigation, Methodology, Project administration, Visualization, Writing – original draft. **J.M. Kotah:** Investigation, Methodology, Writing – review & editing. **J.E. Kuindersma:** Investigation, Methodology. **E. Speijer:** Investigation, Methodology. **A.A.S. van Irsen:** Investigation, Methodology, Writing – review & editing. **S.E. la Fleur:** Conceptualization, Writing – review & editing. **A. Korosi:** Conceptualization, Funding acquisition, Project administration, Supervision, Writing – original draft, Writing – review & editing.

## Appendix A Supplementary data

Fig. S1 Kcal intake per day before the fasting period. **A)** Total caloric intake is higher in fcHFHS fed animals. **B)** Chow intake is lower in fcHFHS exposed animals, and higher in males. **C)** fat intake is higher in females. **D)** sugar intake is not affected by sex or condition. # main effect of diet; $ main effect of sex. Fig. S1
