# Supplementary figures and images for: Adult food choices depend on sex and exposure to early-life stress: Underlying brain circuitry, adipose tissue adaptations and metabolic responses
Source: Neurobiol Stress. 2021 Jun 28;15:100360. doi: 10.1016/j.ynstr.2021.100360 (PMC8264217; doi:10.1016/j.ynstr.2021.100360)

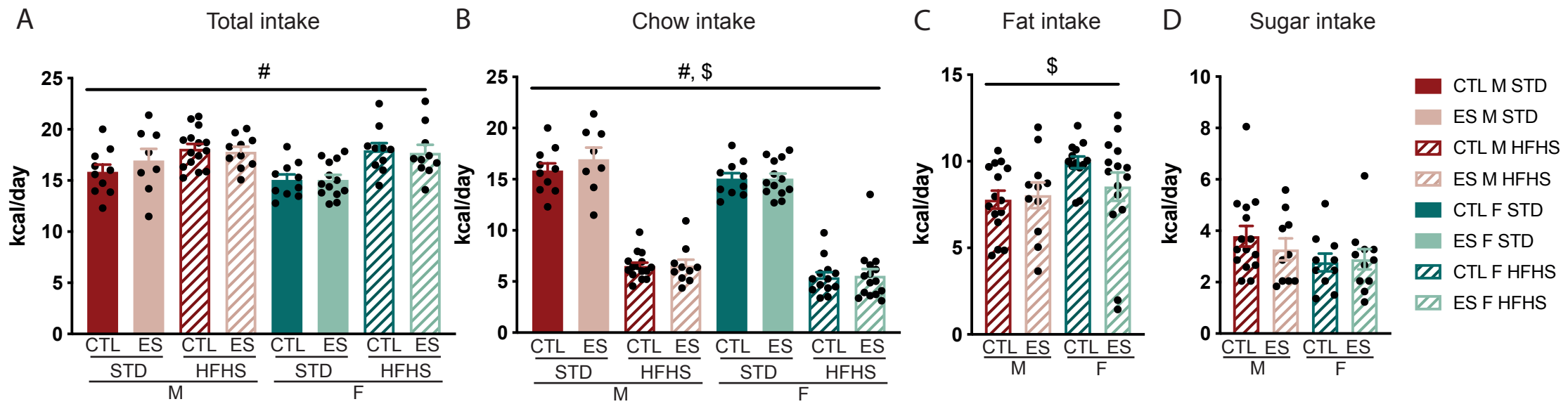

Supplement: Fig. S1 — Kcal intake per day before the fasting period. A) Total caloric intake is higher in fcHFHS fed animals. B) Chow intake is lower in fcHFHS exposed animals, and higher in males. C) fat intake is higher in females. D) sugar intake is not affected by sex or condition. # main effect of diet; $ main effect of sex. [file mmc1.pdf]
